# Age-at-onset in Huntington disease

**DOI:** 10.1371/currents.RRN1258

**Published:** 2011-07-29

**Authors:** Michael Orth, Carsten Schwenke

**Affiliations:** ^*^Department of Neurology, University of Ulm, Ulm, Germany and ^†^Independent Statistical Consultant, SCOSSiS, Germany

## Abstract

Background: In Huntington disease, the accurate determination of age-at-onset is critical to identify modifiers and therapies that aim to delay it.

Methods: Retrospective data from the European Huntington’s Disease Network’s REGISTRY and PREDICT-HD, a longitudinal study in prodromal huntingtin gene expansion mutation carriers. Data (age, gender, CAG repeat length, parent affected, and Unified Huntington’s Disease Rating Scale motor score, total functional capacity) from at least three visits in 423 REGISTRY and 124 PREDICT-HD participants were included. Data based extrapolations of individual age-at-onset using generalized linear mixed models based on individual slopes of motor score or total functional capacity, and predictions using the Langbehn, or Ranen formula, were compared with clinicians’ estimates.

Results: Concordance was best for the observed age-at-onset in PREDICT-HD and the calculated onset using the PREDICT-HD UHDRS longitudinal motor scores. This was superior to the REGISTRY data. For total functional capacity, the investigator’s estimate was 4 years before the data derived age-at-onset. The concordance of predictions of probability of age-at-onset is better with the observed age-at-onset in the PREDICT-HD data (difference in 25%tile -5 to 10 years) than the REGISTRY data (±20 years).

Conclusions: Estimating or predicting age-at-onset in Huntington disease may be inaccurate. It can be useful to 1) add in the manifest population motor score regression derived age-at-onset as additional motor onset and 2) add total functional capacity regression derived age-at-onset for the onset of functional impact of Huntington disease when patients are in mid- to late-stage.

## **INTRODUCTION**

Age-at-onset (AAO) in Huntington disease (HD) describes the point in time when a carrier of the mutated gene develops unequivocal HD signs. The accurate determination of AAO is critical to find factors that modify AAO and to develop and evaluate therapies that aim to delay it. In manifest HD, an autosomal dominant disease with a highly penetrant CAG repeat expansion mutation in the *HTT* gene, ^[1]^ a clinician estimates AAO retrospectively based on information from manifest patients, relatives, and carers. For AAO predictions in the prodromal phase the formula of Langbehn and colleagues uses CAG repeat length and age because of their well known influences on AAO and calculates the time to a predefined degree of probability of manifesting signs of HD.^[2]^ However, CAG repeat length accounts for only about 50-60% of the variability, so other factors not modelled in this formula likely influence AAO.^[3]^ Another formula published by Ranen and colleagues uses CAG repeat length and parental onset age to estimate AAO. ^[4],^ ^[5]^ This may accommodate for some other inherited factors as an advantage over the Langbehn formula. However, it was derived from a small sample of affected parent-child pairs and needs to be validated in larger numbers of patients.^[5]^

The aim of this study is to compare clinicians’ estimates with data-based extrapolations of AAO, and predictions using the Langbehn, or Ranen formula. To this end we used data from two longitudinal observational studies of HD: the European Huntington’s Disease Network’s (EHDN) REGISTRY for preparing the models and PREDICT-HD as independent cohort for testing the new models to predict AAO.^[6],[7]^ REGISTRY enrols participants at any stage of HD including the prodromal phase whereas PREDICT-HD observes *HTT* expansion mutation carriers until they develop unequivocal motor signs of HD.^[6],[7]^ We hypothesised that in the REGISTRY data agreement rates between the clinicians’ estimates of AAO and data based AAO would be lower than in PREDICT-HD since we expected an observed AAO to be more precise than the retrospective estimate of AAO. We further expected that data-based calculations of a motor onset is possible and less variable than clinicians’ estimates of AAO, and, similarly, that it is possible to calculate an onset of functional impairment.  

****

## **METHODS**

### **Participants**

Retrospective data from two ongoing multicentre, longitudinal observational research studies were used: EHDN’s REGISTRY, collecting data in Europe from symptomatic and pre-HD *HTT* mutation expansion carriers (with known CAG repeat length > 36), and PREDICT-HD, a study conducted in the United States, Canada, Europe and Australia in pre-HD (known CAG repeat length > 39).^[6],[7]^ Participants had at least three visits where age, gender, CAG repeat length, parent affected, and clinical data (Unified Huntington’s Disease Rating Scale motor score, total functional capacity) were available.^[8]^ All REGISTRY data were from participants with manifest HD. This was defined as carrying the HD gene mutation and having a motor phenotype that with ≥ 99% certainty was unequivocal for HD (diagnostic confidence of 4 on motor UHDRS).^[8]^ From PREDICT-HD, 124 data sets were included where the investigator had rated a change in the diagnostic confidence to 4.  

Participants gave informed written consent according to the International Conference on Harmonisation-Good Clinical Practice (ICH-GCP) guidelines (http://www.ich.org/LOB/media/MEDIA482.pdf). Ethical approval was obtained from the local ethics committee for each study site contributing to REGISTRY or PREDICT-HD.

### **Data analysis and statistics**

AAO was calculated using generalized linear mixed models based on individual slopes of motor or TFC scores. This means for each participant a linear regression was calculated including intercept and slope of the independent factor across visit dates (Figure 1). Visit was included as random factor. Dependent variables were the date of examination of the motorscore, or TFC, independent variables were "motorscore" or "TFC." The estimates of the regression were then used to extrapolate the AAO by calculating the age for a motorscore of 5, or a TFC of 12, our definitions of manifest disease (Figure 1). 

For AAO predictions, the formula of Langbehn with the predicted probability of signs exceeding 0.6, 0.4 and 0.2 (i.e., "Langb 0.6," "Langb 0.4," and "Langb 0.2"), or the formula of Ranen and colleagues ("Ranen"), was used.^[5]^ We emulated the prodromal stage in our manifest participants by going back in time to when each participant was pre-manifest. We arbitrarily chose an age of 10 years. Secondly, we calculated the disease burden from a participant’s age and CAG repeat length ((CAG_n_-35.5) X age = disease burden) and using the Langbehn formula predicted AAO at an age that corresponded to a disease burden of 200 ("LB DB 0.6").^[9]^

One additional model was based on the CAG repeats, age of affected parent, the motorscore (population-slope) and gender as independent variables and the rater estimate as dependent variable ("Ranen extended"). A second model was estimated analogous to the Ranen formula ("Ranen analog") based on CAG repeats and age of affected parent. Linear regression was used to assess the importance of these factors for the rater estimate. The regression estimates were used to populate the formula.

The pairwise Pearson correlation coefficients were estimated to investigate the linear correlation of each pair of formulae on "AAO." We compared the different models for predicting AAO by calculating agreement rates with Clopper-Pearson 95% confidence intervals between pairs of estimates. If both methods arrived at the same result within a ±5 year bracket the agreement was defined as ‘1’. If the results were more than 5 years different the agreement was defined as ‘0’. For all participants, the agreement rate was expressed as % agreement within accepted range.  

**Figure:**
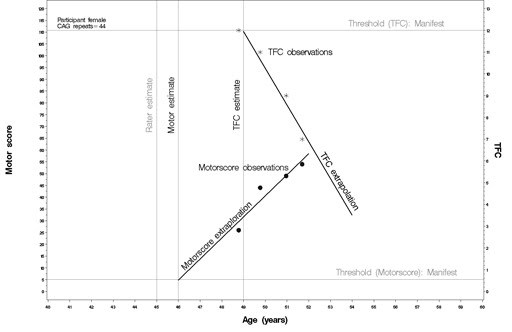

**Figure 1.**

 Illustration of age-at-onset extrapolation from longitudinal UHDRS motor, or total functional capacity (TFC), scores. Linear regression analysis calculates the age for a motorscore of 5 (motor score threshold), or a TFC of 12 (TFC threshold). The vertical lines illustrate the age of the participant at threshold (motor estimate, TFC estimate) and how these calculated onsets compare to the rater estimate. 

## **RESULTS**

### **REGISTRY and PREDICT-HD participants**

Data from 423 REGISTRY participants were included (205 male). The mean CAG repeat expansion was 44 (SD 4), this was similar in men and women. In 193 participants (46%) the gene was inherited from the mother and in 177 (42%) from the father, in 30 (7%) no signs of HD were reported in either parent, and in 23 (5%) the inheritance was unknown for both parents or for at least one parent, where the other parent was not affected. Eight participants (2%) had a juvenile onset (before the age of 20), and 24 (6%) had a late onset of HD above age 60. A motor onset was present in 303, other onset types in 120. The average motor score at enrolment was 35 (out of a possible 124), 158 (37%) participants were in stage 1, 120 (28%) in stage 2, 111 (26%) in stage 3, 30 (7%) in stage 4 and 4 (1%) in stage 5. Patients were first seen by investigators a median of 6 years after the estimated onset. The medium number of visits was 3 (range 3-18).

For PREDICT-HD participants, the median number of visits was 4 (range 2-7). At the visit corresponding to the change in diagnostic confidence to 4 the median motor score was 21.7 (SD 8.6) and the TFC was 11.7 (7.8).

### **AAO extrapolation from longitudinal data**

Sixty-six participants with a negative slope of the motor score were excluded because no individual AAO could be calculated. In the remaining 357 participants, on average the motor score increased linearly by 2.57 points per year. The mean AAO of data based extrapolated motor signs was 47 (range 13-81, SD 11.67). 

Forty-four participants were excluded because the slope of the TFC was positive. This meant no individual TFC onset could be extrapolated based on longitudinal data. In the remaining 379 participants, on average the TFC score decreased linearly by 0.52 points per year. The TFC of the 251 patients in disease stages 1 or 2 decreased by 0.75 points per year compared to 128 patients in disease stages 3, 4, or 5 who on average lost 0.26 points per year. The mean extrapolated AAO was 48 (range 14-81, SD 12, Figure 2). 

The investigator estimated AAO was 3 years before the motorscore calculated AAO (Table 1, Figure 2) with an agreement rate of 0.57 (Table 2). The calculated TFC onset was 4 years later than the investigator’s estimate (Table 1, Figure 2) with an agreement rate of 0.52 (Table 2).

**Figure:**
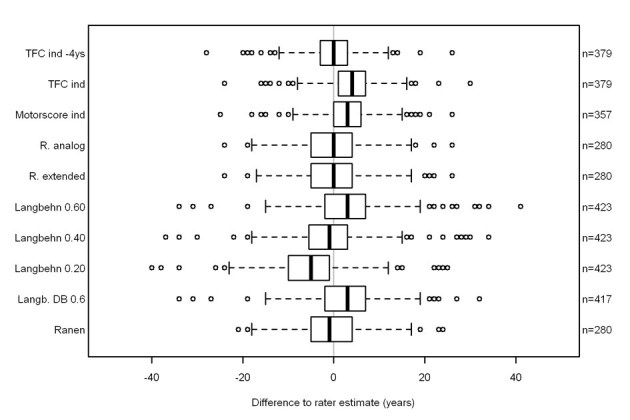

**Figure 2.**

 REGISTRY data: difference to rater estimate. The boxes of the boxplots contain 50% of the data and the median (vertical solid line), the bars indicate the 25%tile and the empty dots outliers. Abbreviations: TFC ind: regression derived TFC age-at-onset. TFC ind – 4 ys: regression derived TFC age-at-onset minus 4 years. Motorscore ind: regression derived motor age-at-onset. R. analog: Ranen analogous. R. extended: Ranen extended.

****

****

**Table 1:**

**Table:** 

**Model**	**N**	**mean**	**SD**	**min**	**Q25**	**median**	**Q75**	**max**
**investigator (rater estimate)**	423	44.08	11.32	10	35.5	44	51.5	77
**Motor score**	357	47.06	11.7	13	38	47	56	81
**TFC score**	379	48	12	14	40	49	56.5	81
**Langbehn age 10**	423	46.82	10.5	23	40	46	54	89
**Langbehn db 200**	417	46.65	10.1	23	40	46	55	74
**Ranen**	280	41.94	7.9	8	38	43	47	61
**Ranen analogous**	280	42.44	8.4	-6	38	44	48	59
**Ranen extended**	280	42.58	8.7	0	38	44	49	60

**Table 1.** REGISTRY data. AAO estimates and data based calculations. Abbreviations: TFC total functional capacity. Langbehn db: Langbehn disease burden.

****

### **Predicting AAO**

The Langbehn formula (LB 06) predicted an AAO of 46.82 (SD 10.5, range 23-89) when 10 years of age; with age at a disease burden of 200, the predicted average AAO was 46.65 (SD 10.14, range 23-74). The agreement rate with the investigator’s estimate was 0.46 (Table 2). In 280 participants (140 women) with the necessary data the Ranen formula predicted an AAO of 41.9 years (SD 7.9, range 8-61). In that group of participants this is similar to the investigator’s estimates of 42 years. The agreement rate was 0.46 (Table 2). 

### **Comparing formulae derived AAO**

In a total of 206 REGISTRY patients (109 women) with complete data the best agreement rate was between the extrapolated AAOs on the longitudinal TFC and motor score data (0.76, Table 2). Using parent AAO and CAG repeats resulted in the following formula that best predicted the investigator’s AAO estimate (Ranen analog): 

AAO=90.3918+0.3293*parent AAO-1.3996*CAG repeats

In a next step we entered all available data into a regression model for the prediction of the investigator’s estimate of AAO (Ranen extended). Motor score, parent AAO, CAG repeats, and gender significantly contributed to the regression model resulting in the following formula: 

AAO=88.7546+0.0430*motorscore+0.3546*parent AAO-1.4311*CAG repeats+1.0124*gender (male=1, female=0). We then entered the dataset of each participant into this formula to arrive at the calculated AAO.  

The data driven formula resembles the Ranen and Ranen analogous formula with high agreement rates (0.97, Table 2) while the agreement rates with other AAO calculations was much lower (Table 2).

We then assessed 145 participants with a motor onset. The findings were similar to the analyses including all participants regardless of major symptom at onset (Table 2); the rater estimated an earlier onset than the AAO from the regression analyses. 

**Table 2.**

**Table:** 

** **	** **	**All participants**				**Participants with motor onset**			
**Formula 1**	**Formula 2**	**N**	**agreement rate**	**95% CI**		**N**	**Agreement rate**	** ** **95% CI**	
Ranen	Langb 06	206	0.50	0.43	0.58	145	0.49	0.41	0.57
Ranen	Langb db 06	206	0.50	0.43	0.58	145	0.49	0.41	0.57
Ranen	R. extended	206	0.97	0.94	0.99	145	0.96	0.91	0.98
Ranen	R. analog	206	0.97	0.94	0.99	145	0.97	0.92	0.99
Ranen	Motor	206	0.35	0.29	0.42	145	0.35	0.27	0.44
Ranen	Rater	206	0.45	0.38	0.52	145	0.44	0.36	0.53
Ranen	TFC	206	0.37	0.31	0.44	145	0.32	0.25	0.41
Langb 06	R. extended	206	0.63	0.56	0.69	145	0.59	0.50	0.67
Langb 06	R. Analog	206	0.65	0.58	0.72	145	0.60	0.52	0.68
Langb 06	Motor	206	0.50	0.43	0.57	145	0.57	0.48	0.65
Langb 06	TFC	206	0.51	0.44	0.58	145	0.55	0.47	0.63
Langb db 06	Langb 06	206	1.00	0.98	1.00	145	1.00	0.97	1.00
Langb db 06	R. extended	206	0.60	0.53	0.67	145	0.55	0.47	0.63
Langb db 06	R. Analog	206	0.64	0.57	0.70	145	0.59	0.50	0.67
Langb db 06	Motor	206	0.51	0.44	0.58	145	0.59	0.50	0.67
Langb db 06	TFC	206	0.51	0.44	0.58	145	0.55	0.47	0.63
R. extended	R. analog	206	1.00	0.98	1.00	145	1.00	0.97	1.00
R. extended	Motor	206	0.41	0.34	0.48	145	0.43	0.35	0.52
R. extended	TFC	206	0.43	0.36	0.50	145	0.40	0.32	0.48
R. analog	Motor	206	0.41	0.34	0.48	145	0.43	0.35	0.51
R. analog	TFC	206	0.44	0.37	0.51	145	0.41	0.33	0.49
Motor	TFC	206	0.76	0.69	0.81	145	0.73	0.65	0.80
Rater	Langb 06	206	0.51	0.44	0.58	145	0.53	0.45	0.61
Rater	Langb db 06	206	0.50	0.43	0.57	145	0.52	0.43	0.60
Rater	R. extended	206	0.49	0.42	0.56	145	0.52	0.44	0.61
Rater	R. analog	206	0.52	0.45	0.59	145	0.54	0.45	0.62
Rater	Motor	206	0.58	0.51	0.65	145	0.61	0.52	0.69
Rater	TFC	206	0.52	0.45	0.59	145	0.52	0.44	0.61

**Table 2.** Agreement rates (± 5years) between different formulae. Agreement rates between pairs of estimates were calculated with Clopper-Pearson 95% confidence intervals. If both methods arrived at the same result within a ±5 year bracket the agreement was defined as ‘1’. If the results were more than 5 years different the agreement was defined as ‘0’. For all participants, the agreement rate was expressed as % agreement within accepted range. Abbreviations: Langb db 06: calculated at an age corresponding to disease burden of 200, age when the predicted probability of signs exceeds 0.6. R. analog: Ranen analogous. R. extended: Ranen extended.  

****

### **PREDICT-HD data**

We assessed how well the rater estimates of AAO, and the calculated AAOs, from the REGISTRY models agreed with the observed AAO in PREDICT-HD participants. Agreement rates were particularly good for the observed AAO in PREDICT-HD and the calculated onset when using the PREDICT-HD UHDRS longitudinal motor scores (0.84, Figure 3). The rater estimated the onset on average three years later than the data derived AAO (s. Figure 3). Next, we compared the formulae to calculate AAO. Agreement rates were very good when ‘Ranen analog’ from REGISTRY data was compared to a similar model derived from PREDICT-HD data (0.98, 95%CI 0.94-1). We then entered the PREDICT-HD data into the REGISTRY data derived formulae to calculate AAO. The agreement rate between rater estimate and formulae derived AAO was best for ‘Ranen analog’ (0.63, Figure 3). As TFC did not decrease over the observation time of the PREDICT-HD participants, the model is not suitable for estimating AAO in this cohort.

We went on to compare agreement rates for predicting AAO when participants were 10 years of age. The best agreement was between the Langbehn formula with 0.2 probability and rater estimate (0.9 (95%CI 0.82-0.95)) whereas it was worse for the Langbehn formula with 0.4 or 0.6 (0.72 (95%CI 0.62-0.8); 0.37 (95%CI 0.28-0.47)), or the Ranen formula (0.57 (95%CI 0.47-0.67)). There was very good agreement between the Ranen formula derived predictions of AAO and the retrospective calculation of AAO with longitudinal data and REGISTRY Ranen analog (0.92, 95%CI 0.86-0.96).

**Figure:**
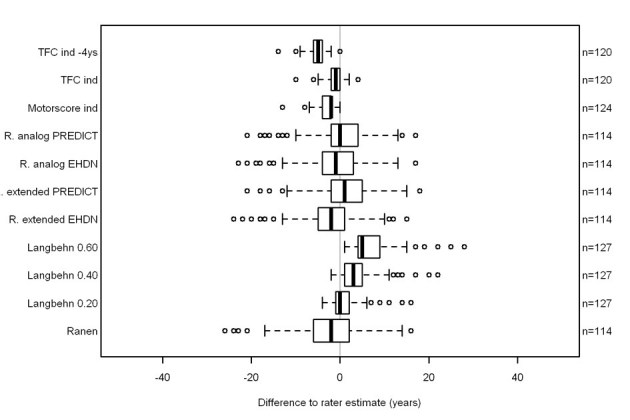

**Figure 3.**

 PREDICT-HD data: difference to rater estimate. The boxes of the boxplots contain 50% of the data and the median (vertical solid line), the bars indicate the 25%tile and the empty dots outliers. Abbreviations: TFC ind: regression derived TFC age-at-onset. TFC ind – 4 ys: regression derived TFC age-at-onset minus 4 years. Motorscore ind: regression derived motor age-at-onset. R. analog: Ranen analogous. R. extended: Ranen extended.

## **DISCUSSION**

The present study assessed how a data derived age-at-onset compares to the rater’s estimate, the commonly used definition of AAO. Using longitudinal data from REGISTRY and PREDICT-HD, two large observational studies, our results suggest that it can be useful to 1) add in the manifest population motor score regression derived AAO as additional motor onset; 2) when patients are in mid- to late-stage HD add TFC regression derived AAO for the onset of functional impact of HD; 3) predictions of AAO suggest a later onset than the actual emergence of unequivocal motor signs of HD.

### **Calculating age-at-onset in manifest HD**

The current gold standard of AAO is a clinician’s estimate integrating data from the patient’s history, collateral history of family or carers and the examination of the patient. We compared a data derived AAO with the estimated AAO. We first used a simple regression analysis of longitudinal UHDRS motor score data and calculated the patient’s age when the motor score was 5 or greater, a cut-off used in TRACK-HD, a large longitudinal multi-centre study. ^[10], [11]^ The AAO from regressing motor scores was 3 years later than the median AAO estimated by REGISTRY investigators. In contrast, in PREDICT-HD the calculated motor onset was 3 years earlier than the observed AAO. The agreement rates between onset in PREDICT-HD data and the calculated motor onset was better than in the REGISTRY data set with a difference of about 20 years in the 25%tile compared with 10 years in PREDICT-HD. In PREDICT-HD participants are enrolled before they have unequivocal motor signs of HD. In contrast, our REGISTRY participants were enrolled when they already had manifest HD. This means that at the REGISTRY enrolment visit the investigator may have had to judge AAO after many years of manifest disease. This AAO estimate would be less accurate than AAO observation in PREDICT-HD. PREDICT-HD investigators, however, may be more conservative with their ratings of conversion to the manifest stage making such a judgement only when clinical signs are unequivocal. A UHDRS motor score of 5 may not equate to such diagnostic certainty, and this may explain why the rater estimate of AAO is later than the data based AAO. In addition, in PREDICT-HD the judgement of conversion from the prodromal to the manifest HD phase rests on the diagnostic confidence of the motor signs. Hence, in REGISTRY an earlier estimated onset than that calculated from longitudinal motor scores may also suggest that investigators are not guided by motor signs alone.

Many studies of genetic modifiers relate their effect to a general onset of HD. It is possible that there are domain specific onset modifiers. Such domain specific modifying effects may be overlooked unless domain specific onsets are defined. Our data suggest that it is possible to use longitudinal motor score data to extrapolate a motor domain onset even in patients with a non-motor onset. This approach has the added advantage that it is based on data collected by certified UHDRS motor scale raters directly examining the patient. This removes some of the variability introduced by judging an onset retrospectively. 

The UHDRS TFC reflects a general impact on the ability to work, handle finances, and the activities of daily living. ^[12]^ The calculated TFC onset was about 4 years later than the rater estimate indicating that it may take a number of years before HD manifestations have a substantial impact on daily life. The good agreement rate between the rater estimate and the calculated ‘TFC onset minus 4 years’ suggests this interval of 4 years is fairly robust and does not depend on the major sign at onset. In the PREDICT-HD data, the TFC does not drop much. Hence, we cannot test the hypothesis that, similar to motor data calculations, the rater estimate in REGISTRY accounts for a substantial part of the observed differences. A ‘TCF onset’ may add an important endpoint based on function. While in the prodromal phase new scales may have to be devised, in midstage HD patients the calculation of an onset of functional impairment using the TFC may help identify the factors that are most relevant to maintain function. ^[13]^

### **Predicting age-at-onset in prodromal HD**

Assuming our manifest participants were prodromal we compared AAO estimations in the REGISTRY data using the Langbehn, or Ranen, formula with the rater estimates of AAO. While the medians agree well, in a substantial proportion of cases the difference was as large as ±20 years. The agreement rates are much better with the PREDICT-HD data with a difference in the 25%tile of about -5 to 10 years between predictions with 20% probability of onset and the rater estimate. Overall, however, the Langbehn formula estimates the AAO later than the raters’ observed AAO. This suggests that unequivocal motor signs of HD manifest earlier, sometimes many years, than predicted using the Langbehn formula.

We next used the data to model predictions of the rater estimate of AAO. Integrating the parent AAO and the individual CAG repeat length resulted in a formula in very good agreement with the Ranen formula. The agreement rates of the Ranen, or Ranen analogous, formula with the rater estimate or the extrapolated AAO revealed a large window of ±20 years. Overall, the difference of the population based analyses, i.e. median, is unbiased, but on individual levels high deviances from the rater estimate were found. This suggests that other factors may also influence the AAO on an individual level that average out on a group level. 

### **Conclusions and limitations**

A methodological limitation relates to the assumption of linear progression. Rates of decline of TFC in our study agree reasonably well with previous data in documenting the rate of decline is slower in the later stages of the disease than in the earlier stages probably reflecting a floor effect. ^[12]^ It also illustrates that the clinical stages of REGISTRY participants are more heterogeneous than those of the PREDICT-HD participants. Progression of HD may not be linear in all stages. Since the population was too small further studies need to investigate rates of progression of HD in large data sets using for example non-linear statistical models. We had to exclude a substantial proportion of participants from the data based extrapolations because the slopes were in the wrong direction. Such individuals present a challenge for the proposed technique of estimating individual onset by extrapolating backwards.

Our results suggest inaccuracies of the concept of a rater estimated AAO. Observing the onset like in PREDICT-HD may result in a more accurate AAO than estimating the AAO from a distance of many years even if experienced HD clinicians use all available information from patients, relatives, and carers. We do not have an objective measure of AAO, or the truth, so we cannot reliably say which of the AAOs we have evaluated is the best. AAO implies it is possible to identify a point in time when HD signs manifest while clinically it would be more appropriate to refer to a transition period of sometimes several years from the prodromal to the manifest stage of HD. Based on our results we suggest to add in manifest HD cohorts a data derived AAO, especially for the motor domain. This may be particularly important when using REGISTRY data where the rater estimate of AAO seems less reliable. For predictions of AAO in the prodromal phase of HD, our data suggest that the Langbehn formula works better than the formula integrating parental AAO. The challenge for the future is to find objective means to define AAO, both predicted and retrospective. REGISTRY and PREDICT-HD, and also TRACK-HD, offer large collections of longitudinal data that can be used to meet this challenge. ^[10]^

****

## **ACKNOWLEDGEMENTS**

This research is supported by the National Institutes for Health, National Institute of Neurological Disorders and Stroke (NS40068) and CHDI Foundation, Inc. We thank the REGISTRY and PREDICT-HD sites and the study participants for their time and effort.

### **REGISTRY Contributors**

**Registry Steering committee:**  A-C Bachoud-Lévi, AR Bentivoglio, I Biunno, R Bonelli, J-M Burgunder, SB Dunnett, JJ Ferreira, OJ Handley, A Heiberg, T Illmann, GB Landwehrmeyer, J Levey, JE Nielsen, M Päivärinta, RAC Roos, A Rojo Sebastián, SJ Tabrizi, W Vandenberghe, C Verellen-Dumoulin, J Zaremba, T Uhrova, J Wahlström

**Information technology:** T Illmann, M Wallner

**Central coordination and Language coordinators:** Katrin Barth, Leonor Correia-Guedes, Ana Maria Finisterra, Monica Bascuñana Garde, Reineke Bos, Sabrina Burg; Daniel Ecker, Olivia J Handley, Christine Held, Kerstin Koppers; Mathilde Laurà, Asunción Martínez Descals, Tim McLean, Tiago Mestre, Sara Minster, Daniela Monza, Jenny Townhill (formerly Naji), Michael Orth, Helene Padieu, Laurent Paterski; Nadia Peppa, Susana Pro Koivisto, Amandine Rialland, Niini Røren (formerly Heinonen) Pavla Šašinková, Patricia Trigo Cubillo, Christine Tritsch, Marlene R van Walsem, Marie-Noelle Witjes-Ané, Elizaveta Yudina (formerly Tarasova), Daniel Zielonka, Eugeniusz Zielonka; Paola Zinzi

**AUSTRIA**

**Graz (LKH Graz, Abteilung für Psychiatrie):** Raphael M. Bonelli; Brigitte Herranhof;  Anna Holl (formerly Hödl); Markus Magnet; Daniela Otti; Annamaria Painold; Karin Reisinger; Monika Scheibl; Helmut Schöggl

**BELGIUM**

**Charleroi (Institut de Pathologie et de Génétique (IPG)):** Pascale Ribaï; Christine Verellen-Dumoulin

**Leuven:** (Universitair Ziekenhuis Gasthuisberg,): Andrea Boogaerts; Wim Vandenberghe;  Dimphna van Reijen

**CZECH REPUBLIC**

**Prague (Extrapyramidové centrum, Neurologická klinika, 1. LF UK a VFN):**  Jiří Klempíř; Jan Roth



**DENMARK**

****

**Copenhagen (Hukommelsesklinikken, Rigshospitalet; Panum Instituttet**): Jørgen E. Nielsen, Lena E. Hjermind, Oda Jakobsen, Jette Stokholm; Lis Hasholt, Anne Nørremølle, Sven Asger Sørensen.

**FINLAND**

**Turku-Suvituuli (Rehabilitation Centre Suvituuli):** Heli Hiivola; Kirsti Martikainen; Katri Tuuha

**Tampere (Terveystalo Healthcare Service Centre); **Maire Santala

**GERMANY**

**Aachen (Universitätsklinikum Aachen, Neurologische Klinik):** Christoph Michael Kosinski; Eva Milkereit ; Daniela Probst; Christian Sass; Johannes  Schiefer; Christiane Schlangen; Cornelius J. Werner

**Berlin (Klinik und Poliklinik für Neurologie - Charité - Universitätsmedizin Berlin)** **:** Harald Gelderblom; Josef Priller; Harald Prüß; Eike Jakob Spruth

**Bochum (Huntington-Zentrum (NRW) Bochum im St. Josef-Hospital):** Jürgen Andrich; Gisa Ellrichmann, Rainer Hoffmann; Christian Prehn, Carsten Saft; Stephan Salmen; Christiane Stamm; Tanja Steiner; Katrin Straßburger 

**Dinslaken (Reha Zentrum in Dinslaken im Gesundheitszentrums Lang):** Herwig Lange

**Dresden (Universitätsklinikum Carl Gustav Carus an der Technischen Universität Dresden, Klinik und Poliklinik für Neurologie):**  Matthias Löhle; Simone Schmidt;  Alexander Storch; Annett Wolz; Martin Wolz

****

#### Freiburg (Universitätsklinik Freiburg, Neurologie): Johann Lambeck, Birgit Zucker

**Hamburg (Universitätsklinikum Hamburg-Eppendorf, Klinik und Poliklinik für Neurologie):** Ute Hidding; Jan Lewerenz; Alexander Münchau; Michael Orth; Jenny Schmalfeld; Lars Stubbe; Simone Zittel

**Heiligenhafen (Psychatrium Heiligenhafen**): Walburgis Heinicke

**Marburg KPP (Klinik für Psychiatrie und Psychotherapie Marburg-Süd): **Bernhard Longinus

**Marburg Uni (Universität Marburg, Neurologie):** Jens Carsten Möller; Ida Rissling

**München (Huntington-Ambulanz im Neuro-Kopfzentrum - Klinikum rechts der Isar der Neurologischen Klinik und Poliklinik der Technischen Universität München): **Alexander Peinemann; Michael Städtler; Adolf Weindl

**Münster (Universitätsklinikum Münster, Klinik und Poliklinik für Neurologie):** Stefan Bohlen; Eva Hölzner; Herwig Lange; Ralf Reilmann

****

**Taufkirchen (Isar-Amper-Klinikum - Klinik Taufkirchen (Vils)):** Antonie Beister; Matthias Dose; Kathrin Hammer; Janina Kieni; Gabriele Leythaeuser; Ralf Marquard; Tina Raab; Sven Richter; Amina Selimbegovic-Turkovic; Caroline Schrenk; Michele Schuierer; Alexandra Wiedemann 

**Ulm (Universitätsklinikum Ulm, Neurologie):** Katrin Barth; Andrea Buck; Julia Connemann; Daniel Ecker; Carolin Eschenbach; Christine Held; Bernhard Landwehrmeyer; Franziska Lezius; Anke Niess; Michael Orth, Sigurd Süßmuth, Sonja Trautmann; Patrick Weydt

**ITALY**

**Bari (Dipartimento di Scienze Neurologiche e Psichiatriche Universita' di Bari):**  Claudia Cormio; Olimpia Difruscolo; Vittorio Sciruicchio; Claudia Serpino; Marina de Tommaso

**Florence (Dipartimento di Scienze Neurologiche e Psichiatriche Universita' degli Studi di Firenze-Azienda Ospedaliera Universitaria Careggi):** Elisabetta Bertini; Elena Ghelli, Andrea Ginestroni, Francesca Massaro, Claudia Mechi; Marco Paganini; Silvia Piacentini; Silvia Pradella; Anna Maria Romoli; Sandro Sorbi

**Genoa (Dipartimento di Neuroscienze, Oftalmologia e Genetica (DiNOG)  Università di Genova):** Giovanni Abbruzzese; Monica Bandettini di Poggio; Giovanna   Ferrandes; Paola Mandich; Roberta Marchese

**Milan (Fondazione IRCCS Istituto Neurologico Carlo Besta**):   Alberto Albanese; Daniela Di Bella;  Stefano Di Donato; Cinzia Gellera; Silvia Genitrini; Caterina Mariotti; Daniela Monza; Lorenzo Nanetti; Dominga Paridi; Paola Soliveri; Chiara Tomasello

**Naples (Dipartimento di Scienze Neurologiche  Azienda Ospedaliera Universitaria Federico II ):** Giuseppe De Michele; Luigi Di Maio; Carlo Rinaldi; Cinzia Valeria Russo, Elena Salvatore; Tecla Tucci

**Pozzilli (IS) (Centro di Neurogenetica e Malattie Rare – IRCCS Neuromed**) Francesca Elifani, Sara Orobello, Silvia Alberti, Annamaria Griguoli, Enrico Amico, Annunziata De Nicola, Tiziana Martino; Ferdinando Squitieri 

**Rome (Istituto di Neurobiologia e Medicina Molecolare & Istituto di Scienze e Tecnologie della Cognizione /CNR;  Istituto di Neurologia Università Cattolica del Sacro Cuore):** Anna Rita Bentivoglio;  Claudio Catalli; Raffaella Di Giacopo; Alfonso Fasano; Marina Frontali; Arianna Guidubaldi; Tamara Ialongo; Gioia Jacopini; Giovanna Loria; Carla Piano; Piccininni Chiara; Davide Quaranta; Silvia Romano; Francesco Soleti; Maria Spadaro; Paola Zinzi

**NETHERLANDS**



**Enschede (Medisch Spectrum Twente):** Monique S.E. van Hout; Jeroen P.P. van Vugt; A. Marit de Weert



**Groningen (Polikliniek Neurologie**): J.J.W. Bolwijn; M. Dekker; K.L. Leenders;  J.C.H. van Oostrom

**Leiden (Leiden University Medical Centre (LUMC**)): Reineke Bos; Eve M. Dumas; Caroline K. Jurgens; Simon J. A. van den Bogaard;  Raymund A.C. Roos; Ellen P. ‘t Hart; Marie-Noëlle Witjes-Ané

**Nijmegen (Universitair Medisch Centrum St. Radboud, Neurology**): Berry Kremer; C.C.P. Verstappen

**NORWAY**

**Oslo University Hospital (Rikshospitalet, Dept. of Medical Genetics and Dep. of Neurology):** Arvid Heiberg; Marleen R van Walsem; Jan Frich; Olaf Aaserud; Raghild Wehus 

**Oslo** **University Hospital** **(Ulleval**, **Dept of Medical Genetics and Department)**: Kathrine Bjørgo; Madelein Fannemel; Per Gørvell; Eirin Lorentzen; Susana Pro Koivisto; Lars Retterstøl; 

**Trondheim (St. Olavs Hospital):** Inga Bjørnevoll; Sigrid Botne Sando

****

**POLAND**

****

**Gdansk** **(St. Adalbert Hospital, Gdansk;  Medical University of Gdansk, Neurological and Psychiatric Nursing  Dpt.):** Emilia Sitek; Jaroslaw Slawek; Witold Soltan.

****

**Katowice (Silesian Medical University Katowice):** Magdalena Boczarska-Jedynak; Barbara Jasinska-Myga; Gregorz Opala; Gabriela Kłodowska - Duda.

**Krakow (Krakowska Akademia Neurologii):** Krzysztof Banaszkiewicz; Andrzej Szczudlik; Monika Rudzińska; Magdalena Wójcik; Małgorzata Dec; Malgorzata Krawczyk.

**Poznan (Medical University of Poznań):** Anna Bryl; Anna Ciesielska; Aneta Klimberg; Jerzy Marcinkowski; Justyna Sempołowicz; Daniel Zielonka; Husam Samara.

**Warsaw-MU (Medical University of Warsaw, Neurology):** Piotr Janik; Anna Gogol, Hubert Kwiecinski; Zygmunt Jamrozik

**Warsaw-IPiN (Institute of Psychiatry and Neurology Dep. of Genetics, Dep. of Neurology**): Jakub Antczak; Katarzyna Jachinska; Maria Rakowicz; Przemyslaw Richter; Danuta Ryglewicz; Grzegorz Witkowski; Elzbieta Zdzienicka; Jacek Zaremba; Anna Sułek, Wioletta Krysa.

**PORTUGAL**

****

**Lisbon- (Hospital de Santa Maria; Neurological Clinical Research Unit, Instituto de Medicina Molecular):** Tiago Mestre; Leonor Correia-Guedes; Miguel Coelho; Joaquim J Ferreira

**Lisbon- (Hospital Fernando da Fonseca) :** Ângela Timóteo; Cristina Costa

**Porto- (** **Hospital de São João)** **:** Miguel Gago; Carolina Garrett; Maria Rosália Guerra.

**SPAIN**

**Badajoz (Hospital Infanta Cristina):** Carmen Durán Herrera; Patrocinio Moreno Garcia

**Granada (Hospital Universitario San Cecilio, Neurología**): Francisco Barrero; Blas Morales 

****

**Burgos (Servicio de Neurología Hospital General Yagüe):** Esther Cubo; Natividad Mariscal; Jesús Sánchez

**Fuenlabrada (Hospital Universitario de Fuenlabrada): **Fernando Alonso-Frech; Maria Rabasa Perez

**Madrid-Clinico (Hospital Clínico Universitario San Carlos):** María Fenollar; Rocío García-Ramos García; Purificacion Pin Quiroga; Susana Vázquez Rivera; Clara Villanueva 

****

**Madrid RYC (Hospital Ramón y Cajal, Neurología):** Mónica Bascuñana; Marta Fatás Ventura; Guillermo García Ribas; Garcia Riva, Justo García de Yébenes; José Luis López – Sendón Moreno, Patricia Trigo Cubillo 

**Madrid FJD (Madrid-Fundación Jiménez Díaz):** Pedro J García Ruíz, Asunción Martínez-Descals, María José Saiz Artiga; Vicenta Sánchez

**Barcelona-Hospital Mútua de Terrassa:** Miquel Aguilar Barbera; Dolors Badenes Guia; Laura Casas Hernanz ; Judit López Catena; Ana Rojo Sebastián, Pilar Quiléz Ferrer; Gemma Tome Carruesco

**Barcelona-Bellvitge (Hospital Universitari de Bellvitge):** Jordi Bas; Núria Busquets

Matilde Calopa

**Barcelona- Hospital Clinico:** Maria Teresa Buongiorno; Esteban Muñoz

**Barcelona-Merced (Hospital Mare de Deu de La Merced):** Marina Dalmau Elorza; Cristóbal Díez-Aja López; Santiago Durán-Sindreu Terol; Misericordia Floriach Robert; Belén Garzón Ruíz; Ana González Casado; Isabel Haro Martínez; Celia Mareca Viladrich; Regina Pons i Càrdenas; Elvira Roca; Joan Roig Llesoy; Jesús Miguel Ruiz Idiago; Mar Ruíz Vergara; Socorro Soriano García; Antonio Villa Riballo

**Palma (Hospital Son Dureta):** Aranzazú Gorospe; Inés Legarda; Penelope Navas Arques; María José Torres Rodríguez, Barbara Vives

**Pamplona (Hospital Virgen del Camino, Medical Genetic):** Itziar Gaston; Maria A. Ramos-Arroyo, Maria Dolores Martinez-Jaurrieta

**SWEDEN**

**Stockholm (Karolinska University Hospital):** Sven E Pålhagen; Martin Paucar; Per Svenningsson; Tina Walldén Reza-Soltani; Arja Höglund; Britta Sandström 

****

**Stockholm-Ersta (NeuroHealth Consulting Sweden HB, Karolinska Institute):** Joakim Tedroff ; Mona Esmaeilzadeh; Elisabeth Winnberg

**Uppsala (Uppsala University Hospital):** Anders Johansson; Leif Wiklund; Camilla Ekwall; Marie-Louise Göller; Yvonne Björn

**SWITZERLAND**

**Bern:** Jean-Marc Burgunder; Yvonne Burgunder; Yanik Stebler **(Neurologische Klinik des Inselspitals);** ; Alain Kaelin; Irene Romero; Michael Schüpbach; Sabine Weber Zaugg **(Zentrum für Bewegungsstörungen, Neurologische Klinik und Poliklinik)**

**U.K.**

****

**Aberdeen (NHS Grampian, Clinical Genetics Centre):** Roisin Jack; Kirsty Matheson; Zosia Miedzybrodzka; Daniela Rae; Sheila Simpson; Fiona Summers; Alexandra Ure

**Birmingham** **(The Barberry Centre, Dept of Psychiatry**): Adrienne Curtis; Jenny de Souza (Keylock); Hugh Rickards; Jan Wright

**Cambridge (Cambridge Centre for Brain Repair, Forvie Site**): Matthew Armstrong, Roger A. Barker; Deidre O’Keefe, Anna Di Pietro; Kate Fisher; Anna Goodman; Susan Hill; Ann Kershaw; Sarah Mason; Nicole Paterson; Rachel Swain, Lucy Raymond 

**Cardiff (The Institute of Medical Genetics, University Hospital of Wales):** Monica Busse, Stephen Dunnett, Catherine Clenaghan, Ruth Fullham, Sarah Hunt, Lesley Jones, Una Jones, Hanan Khalil, Sara Minster, Michael Owen, Kathleen Price, Jenny Townshill,  Anne Rosser

**Edinburgh (Molecular Medicine Centre, Western General Hospital, Department of Clinical Genetics):** Maureen Edwards; Teresa Hughes (Scottish Huntington´s Association); Marie McGill; Pauline Pearson; Mary Porteous; Paul Smith (Scottish Huntington´s Association); Adam Zeman

**Fife (Scottish Huntington's Association Whyteman's Brae Hospital):** Peter Brockie; Jillian Foster; Nicola Johns; Sue McKenzie, Jean Rothery, Gareth Thomas, Shona Yates

**Glasgow** **(Abercromby Center):** Joanne Miller; Stuart Ritchie

****

**Gloucester** **(Department of Neurology Gloucestershire Royal Hospital):** Liz Burrows; Amy Fletcher; Alison Harding, Fiona Laver; Mark Silva; Aileen Thomson

**Hull** **(Castle Hill Hospital):** Peter Burns; Carol Chu; Carole Evans; Stephanie Hamer; Ivana Markova; Julie Miller; Ashok Raman

**Leeds (Chapel Allerton Hospital, Department of Clinical Genetics):** Kathy Barnes; Carol Chu; Emma Hobson; Stuart Jamieson; Ivana Markova; Jenny Thomson; Jean Toscano; Sue Wild; Pam Yardumian 

**Leicester (Leicestershire Partnership Trust, Mill Lodge):** Colin Bourne; Carole Clayton; Heather Dipple; Jackie Clapton, Janet Grant; Diana Gross; Caroline Hallam; Julia Middleton; Ann Murch, Dawn Patino

**London (Guy's Hospital):** Thomasin Andrews; Andrew Dougherty; Fred Kavalier; Charlotte Golding; Alison Lashwood; Dene Robertson; Deborah Ruddy; Anna Whaite 

****

**London** **(St. Georges-Hospital):** Michael Patton, Maria Peterson; Sarah Rose

**London (The National Hospital for Neurology and Neurosurgery**): Thomasin Andrews; Stefania Bruno; Charlotte Golding; Nayana Lahiri; Marianne Novak; Aakta Patel; Elisabeth Rosser; Sarah Tabrizi; Rachel Taylor; Thomas Warner; Edward Wild

****

**Manchester (Genetic Medicine, University of Manchester, Manchester Academic Health Sciences Centre and Central Manchester University Hospitals NHS Foundation Trust):** Natalie Arran, Jenny Callaghan, David Craufurd, Ruth Fullam, Liz Howard, Susan Huson, Lucy Partington-Jones, Julie Snowden, Andrea Sollom, Jennifer Thompson, Cheryl Stopford, Nichola Verstraelen (formerly Ritchie), Leann Westmoreland

**Oxford** **(Oxford Radcliffe Hospitals NHS Trust):** Andrea H Nemeth; Gill Siuda

**Plymouth** **(Heathleigh Unit, Mount Gould Hospital):** David Harrison; Max Hughes; Andrew Parkinson; Beverley Soltysiak

**Sheffield (The Royal Hallamshire Hospital– Sheffield Children’s Hospital):** Oliver Bandmann; Alyson Bradbury, Paul Gill, Helen Fairtlough, Kay Fillingham, Isabella Foustanos; Kirsty O’Donovan; Nadia Peppa, Katherine Tidswell, Oliver Quarrell

****

****

**PREDICT-HD Investigators, Coordinators, Motor Raters, Cognitive Raters**

*Active: September 2009 – August 2010*

Thomas Wassink, MD, Stephen Cross, BA, Nicholas Doucette, BA, Mycah Kimble, BA, Patricia Ryan, MSW, LISW, MA, Jessica Wood, MD, PhD, Eric A. Epping, MD, PhD, and Leigh J. Beglinger, PhD (University of Iowa, Iowa City, Iowa, USA);

Edmond Chiu, MD, Olga Yastrubetskaya, PhD, Joy Preston, Anita Goh, D.Psych, Chathushka Fonseka, and Liz Ronsisvalle (St. Vincent’s Hospital, The University of Melbourne, Kew, Victoria, Australia); 

Phyllis Chua, MD, and Angela Komiti, BS, MA (The University of Melbourne, Royal Melbourne Hospital, Melbourne, Australia)

Lynn Raymond, MD, PhD, Rachelle Dar Santos, BSc, and Joji Decolongon, MSC, CCRP (University of British Columbia, Vancouver, British Columbia, Canada); 

Adam Rosenblatt, MD, Christopher A. Ross, MD, PhD, Barnett Shpritz, BS, MA, OD, and Claire Welsh (Johns Hopkins University, Baltimore, Maryland, USA); 

William M. Mallonee, MD, Greg Suter, BA, and Judy Addison (Hereditary Neurological Disease Centre, Wichita, Kansas, USA); 

Ali Samii, MD, and Alma Macaraeg, BS (University of Washington and VA Puget Sound Health Care System, Seattle, Washington, USA); 

Randi Jones, PhD, Cathy Wood-Siverio, MS, Stewart A. Factor, DO, and Claudia Testa, MD, PhD (Emory University School of Medicine, Atlanta, Georgia, USA); 

Roger A. Barker, BA, MBBS, MRCP, Sarah Mason, BSC, Anna Goodman, PhD, Rachel Swain, BA, and Anna DiPietro (Cambridge Centre for Brain Repair, Cambridge, UK);

Elizabeth McCusker, MD, Jane Griffith, RN, Clement Loy, MD, David Gunn, BS, and Linda Stewart, RN (Westmead Hospital, Sydney, Australia); 

Bernhard G. Landwehrmeyer, MD, Michael Orth MD, PhD, Sigurd Süβmuth, MD, RN, Katrin Barth, RN, and Sonja Trautmann, RN (University of Ulm, Ulm, Germany);

Kimberly Quaid, PhD, Melissa Wesson, MS, and Joanne Wojcieszek, MD (Indiana University School of Medicine, Indianapolis, IN);

Mark Guttman, MD, Alanna Sheinberg, BA, and Irita Karmalkar, BSc (Centre for Addiction and Mental Health, University of Toronto, Markham, Ontario, Canada); 

Susan Perlman, MD and Arik Johnson, PsyD (University of California, Los Angeles Medical Center, Los Angeles, California, USA); 

Michael D. Geschwind, MD, PhD, Jon Gooblar, BA, and Gail Kang, MD (University of California San Francisco, California, USA);

Tom Warner, MD, PhD, Maggie Burrows, RN, BA, Marianne Novak, MD,Thomasin Andrews, MD, BSC, MRCP, Elisabeth Rosser, MBBS, FRCP, and Sarah Tabrizi, BSC, PhD (National Hospital for Neurology and Neurosurgery, London, UK); 

Anne Rosser, MD, PhD, MRCP, Kathy Price, RN, and Sarah Hunt, BSc (Cardiff University, Cardiff, Wales, UK); 

Frederick Marshall, MD, Amy Chesire, LCSW-R, MSG, Mary Wodarski, BA, and Charlyne Hickey, RN, MS (University of Rochester, Rochester, New York, USA); 

Oksana Suchowersky, MD, FRCPC, Sarah Furtado, MD, PhD, FRCPC, and Mary Lou Klimek, RN, BN, MA (University of Calgary, Calgary, Alberta, Canada); 

Peter Panegyres, MB, BS, PhD, Elizabeth Vuletich, BSC, Steve Andrew, and Rachel Zombor, MPSYC (Neurosciences Unit, Graylands, Selby-Lemnos & Special Care Health Services, Perth, Australia); 

Joel Perlmutter, MD, Stacey Barton, MSW, LCSW, and Amy Schmidt (Washington University, St. Louis, Missouri, USA); 

Zosia Miedzybrodzka, MD, PhD, Sheila A. Simpson, MD, Daniela Rae, RN, and Mariella D’Alessandro, PhD (Clinical Genetics Centre, Aberdeen, Scotland, UK); 

David Craufurd, MD, Ruth Fullam, BSC, and Elizabeth Howard, MD (University of Manchester, Manchester, UK); 

Pietro Mazzoni, MD, PhD, Karen Marder, MD, MPH, and Paula Wasserman, MA (Columbia University Medical Center, New York, New York, USA); 

Rajeev Kumar, MD and Diane Erickson, RN (Colorado Neurological Institute, Englewood, Colorado, USA); 

Vicki Wheelock, MD, Terry Tempkin, RNC, MSN, Nicole Mans, BA, MS, and Kathleen Baynes, PhD (University of California Davis, Sacramento, California, USA); 

Joseph Jankovic, MD, Christine Hunter, RN, CCRC, and William Ondo, MD (Baylor College of Medicine, Houston, Texas, USA); 

Justo Garcia de Yebenes, MD, Monica Bascunana Garde, Marta Fatas, BA,  and Asuncion Martinez-Descales (Hospital Ramón y Cajal, Madrid, Spain); 

Wayne Martin, MD, Pamela King, BScN, RN, and Satwinder Sran, BSC (University of Alberta, Edmonton, Alberta, Canada); 

Anwar Ahmed, PhD, Stephen Rao, PhD, Christine Reece, BS, Janice Zimbelman, PhD, PT, Alexandra Bea, BA, Emily Newman, BA, and Alex Bura, BA (Cleveland Clinic Foundation, Cleveland, Ohio, USA).

**Steering Committee**

Jane Paulsen, PhD, Principal Investigator, Eric A. Epping, MD, PhD, Hans Johnson, PhD, Megan Smith, PhD, Janet Williams, PhD, RN, FAAN, Leigh Beglinger, PhD, James Mills, MS (University of Iowa Hospitals and Clinics, Iowa City, IA); Elizabeth Aylward, PhD (Seattle Children's Research Institute, WA); Kevin Biglan, MD (University of Rochester, Rochester, NY); Blair Leavitt, MD (University of British Columbia, Vancouver, BC, Canada); Marcy MacDonald, PhD (Massachusetts General Hospital); Martha Nance, MD (Hennepin County Medical Center, Minneapolis, MN); and Cheryl Erwin, JD, PhD (University of Texas Medical School at Houston).

**Scientific Sections**

**Bio Markers: **Blair Leavitt, MDCM, FRCPC (Chair) and Michael Hayden, PhD (University of British Columbia); Stefano DiDonato, MD (Neurological Institute “C. Besta,” Italy); Ken Evans, PhD (Ontario Cancer Biomarker Network); Wayne Matson, PhD (VA Medical Center, Bedford, MA); Asa Peterson, MD, PhD (Lund University, Sweden), Sarah Tabrizi, PhD (National Hospital for Neurology and Neurology and Neurosurgery, London); Beth Borowsky, PhD (CHDI); Andrew Juhl, BS, James Mills, MS, Kai Wang, PhD (University of Iowa); and David Weir, BSc (University of British Columbia).

**Brain:**  Jean Paul Vonsattell, PhD (Chair), and Carol Moskowitz, ANP, MS (Columbia University Medical Center); Anne Leserman, MSW, LISW, Lynn Schaul, BA, and Stacie Vik, BA (University of Iowa).

**Cognitive:** Deborah Harrington, PhD (Chair), Gabriel Castillo, BS, Jessica Morison, BS, and Jason Reed, BS (University of California, San Diego), Michael Diaz, PhD, Ian Dobbins, PhD, Tamara Hershey, PhD, Erin Foster, OTD, and Deborah Moore, BA (Washington University Cognitive Science Battery Development); Holly Westervelt, PhD (Chair, Quality Control and Training, Alpert Medical School of Brown University), Jennifer Davis, PhD, and Geoff Tremont, PhD, MS (Scientific Consultants, Alpert Medical School of Brown University); Megan Smith, PhD (Chair, Administration), David J. Moser, PhD, Leigh J. Beglinger, PhD, Kelly Rowe, and Danielle Theriault, BS (University of Iowa); Carissa Gehl, PhD (VA Medical Center, Iowa City, IA); Kirsty Matheson (University of Aberdeen); Karen Siedlecki, PhD (Fordham University); Marleen Van Walsem (EHDN); Susan Bonner, BA, Greg Elias, BA, and Melanie Faust, BS (Rhode Island Hospital); Beth Borowski, PhD (CHDI); Noelle Carlozzi (University of Michigan); Kevin Duff, PhD (University of Utah); Nellie Georgiou-Karistianis (St. Vincent’s Hospital, The University of Melbourne, Australia); Julie Stout, PhD (Monash University, Melbourne, Australia); Herwig Lange (Air-Rahazentrum); and Kate Papp (University of Connecticut).

**Functional**: Janet Williams, PhD (Chair), Leigh J. Beglinger, PhD, Anne Leserman, MSW, LISW, Eunyoe Ro, MA, Lee Anna Clark, Nancy Downing, Joan Laing, PhD, Kristine Rees, BA, and Stacie Vik, BA (University of Iowa); Rebecca Ready, PhD (University of Massachusetts); Anthony Vaccarino, PhD (Ontario Cancer Biomarker Network); Sarah Farias, PhD (University of California, Davis); Noelle Carlozzi, PhD (University of Michigan); and Carissa Gehl, PhD (VA Medical Center, Iowa City, IA).

**Genetics:** Marcy MacDonald, PhD (Co-Chair), Jim Gusella, PhD, and Rick Myers, PhD (Massachusetts General Hospital); Michael Hayden, PhD (University of British Columbia); Tom Wassink, MD (Co-Chair) Eric A. Epping, MD, PhD, Andrew Juhl, BA, James Mills, MS, and Kai Wang, PhD (University of Iowa); Zosia Miedzybrodzka, MD, PhD (University of Aberdeen); and Christopher Ross, MD, PhD (Johns Hopkins University).

**Imaging** **:** ***Administrative:*** Ron Pierson, PhD (Chair), Kathy Jones, BS, Jacquie Marietta, BS, William McDowell, AA, Greg Harris, BS, Eun Young Kim, MS, Hans Johnson, PhD, and Thomas Wassink, MD (University of Iowa); John Ashburner, PhD (Functional Imaging Lab, London); Steve Potkin, MD (University of California, Irvine); and Arthur Toga, PhD (University of California, Los Angeles).

***Striatal:***Elizabeth Aylward, PhD (Chair, Seattle Children's Research Institute).

***Surface Analysis:*** Peg Nopoulos, MD (Chair), and Eric Axelson, BSE (University of Iowa).

***Shape Analysis:*** Christopher A. Ross (Chair), MD, PhD, Michael Miller, PhD, and Sarah Reading, MD (Johns Hopkins University); Mirza Faisal Beg, PhD (Simon Fraser University).

***DTI:***Vincent A. Magnotta, PhD (Chair, University of Iowa); Karl Helmer, PhD (Massachusetts General Hospital); Kelvin Lim, MD (University of Ulm, Germany); Mark Lowe, PhD (Cleveland Clinic); Sasumu Mori, PhD (Johns Hopkins University); Allen Song, PhD (Duke University); and Jessica Turner, PhD (University of California, Irvine). 

***fMRI:***Steve Rao, PhD (Chair), Erik Beall, PhD, Katherine Koenig, PhD, Michael Phillips, MD, Christine Reece, BS, and Jan Zimbelman, PhD, PT (Cleveland Clinic); and April Bryant (University of Iowa).

**Motor**: Kevin Biglan, MD (University of Rochester), Karen Marder, MD (Columbia University), and Jody Corey-Bloom, MD, PhD (University of California, San Diego) all Co-Chairs; Michael Geschwind, MD, PhD (University of California, San Francisco); Ralf Reilmann, MD and Zerka Unds (Muenster, Germany); and Andrew Juhl, BS (University of Iowa).

**Psychiatric** **:** Eric A. Epping, MD, PhD (Chair), Nancy Downing, RN, MSN, Jess Fiedorowicz, MD, Robert Robinson, MD, Megan Smith, PhD, Leigh Beglinger, PhD, James Mills, MS, Kristine Rees, BA, Adam Ruggle, Stacie Vik, BA, Janet Williams, PhD, Dawei Liu, PhD, David Moser, PhD, and Kelly Rowe (University of Iowa); Karen Anderson, MD (University of Maryland); David Craufurd, MD (University of Manchester); Mark Groves, MD (Columbia University); Anthony Vaccarino, PhD and Ken Evans, PhD (Ontario Cancer Biomarker Network); Hugh Rickards, MD (Queen Elizabeth Psychiatric Hospital); Eric van Duijn, MD (Leiden University Medical Center, Netherlands); Irina Antonijevic, MD, PhD, and Joseph Giuliano (CHDI); Phyllis Chua (The University of Melbourne, Royal Melbourne Hospital); and Kimberly Quaid, PhD (Indiana University School of Medicine).

**Core Sections**

**Statistics:** James Mills, MEd, MS, Dawei Liu, PhD, Jeffrey Long, PhD, Wenjing Lu, Kai Wang, PhD, and Ying Zhang, PhD (University of Iowa).

**Recruitment/Retention:** Martha Nance, MD (Chair, University of Minnesota); Anne Leserman, MSW, LISW, Nicholas Doucette, BA, Mycah Kimble, BA, Patricia Ryan, MSW, LISW, MA, Kelli Thumma, BA, Elijah Waterman, BA, and Jeremy Hinkel, BA (University of Iowa).

**Ethics:** Cheryl Erwin, JD, PhD, (Chair, McGovern Center for Health, Humanities and the Human Spirit); Eric A. Epping, MD, PhD Janet Williams, PhD, Nicholas Doucette, BA, Anne Leserman, MSW, LISW, James Mills, MS, Lynn Schaul, BA, and Stacie Vik, BA (University of Iowa); Martha Nance, MD (University of Minnesota); and Lisa Hughes, MEd (University of Texas Medical School at Houston).

**IT/Management:** Hans Johnson, PhD (Chair), R.J. Connell, BS, Karen Pease, BS, Ben Rogers, BA, BSCS, Jim Smith, AS, Shuhua Wu, MCS, Roland Zschiegner, Erin Carney, Bill McKirgan, Mark Scully, and Ryan Wyse (University of Iowa); Jeremy Bockholt (AMBIGroup).

**Program Management**

**Administrative:**Chris Werling-Witkoske (Chair), Karla Anderson, BS, Kristine Bjork, BA, Ann Dudler, Jamy Schumacher, Sean Thompson, BA, Leann Davis, Machelle Henneberry, Greg Ennis, MA, and Stacie Vik, BA (University of Iowa).

**Financial:**Steve Blanchard, MSHA, Kelsey Montross, BA, and Phil Danzer (University of Iowa).

**Documentation of author roles**

1.       Research project: A. Conception, B. Organization, C. Execution;

2.       Statistical Analysis: A. Design, B. Execution, C. Review and Critique;

3.       Manuscript Preparation: A. Writing of the first draft, B. Review and Critique;

Michael Orth: 1A,B,C;  2A,C; 3A,B

Carsten Schwenke: 1A,B,C; 2A,B,C; 3B

**Full financial disclosure of the previous 12 months:** M Orth’s salary at the University of Ulm is funded by CHDI, Inc.. The Huntington disease study site in Ulm has received compensation for taking part in the following clinical trials and observational studies: ACR16 (Neurosearch), Horizon (Medivation), REGISTRY (European Huntington’s Disease Network), PREDICT-HD (Huntington Study Group). Carsten Schwenke receives honoraria as freelance statistician from different pharmaceutical companies.

**Competing interests**

The authors report no competing interests.

****
